# Broadband Metallic Planar Microlenses in an Array: the Focusing Coupling Effect

**DOI:** 10.1186/s11671-016-1333-9

**Published:** 2016-02-27

**Authors:** Yiting Yu, Ping Wang, Yechuan Zhu, Jinshuai Diao

**Affiliations:** Key Laboratory of Micro/Nano Systems for Aerospace, Ministry of Education, Northwestern Polytechnical University, Xi’an, 710072 China; Key Laboratory of Micro- and Nano-Electro-Mechanical Systems of Shaanxi Province, Northwestern Polytechnical University, Xi’an, 710072 China

**Keywords:** Metallic planar microlenses, Microlens arrays, Nanosphere lithography, Focusing coupling

## Abstract

The microlens arrays (MLAs) are widely utilized for various applications. However, when the lens size and the spacing between two adjacent microlenses are of the length scale of the working wavelength, the diffraction effect plays a vital role in the final focusing performance. We suggest a kind of broadband metallic planar microlenses, based on which the ultra-compact microlens arrays are also constructed. The focusing coupling effect revealing for such devices is then investigated in detail by using the finite-difference time-domain (FDTD) method, with the emphasis on the changing spacing between adjacent microlenses, the working wavelength, the diameter of microlenses, and the array size. The results show that a larger spacing, a larger lens size, a shorter wavelength, or a smaller array scale can lead to a weaker focusing coupling effect. This research provides an important technological reference to design an array of metallic planar microlenses with the well-controlled focusing performance.

## Background

The microlens arrays (MLAs), as a kind of very important optical elements, are widely used in various fields, such as charge-coupled devices (CCDs) [1], displays [2, 3], LED lighting [4], solar concentrators [5], and photolithography [6]. The miniaturization of the MLAs is essential for the development of modern solid-state imaging sensors and other opto-electronic applications. The focusing capabilities of conventional, dielectric-based MLAs, however, deteriorate as their physical dimensions approach the wavelength.

In the last decade, the field of plasmonics, also named as the metal optics, has achieved an explosive development for various potential applications, due to its capabilities to route and manipulate light at the nanometer length scale [7–12], as well as the increasing maturity of the available nanofabrication techniques [13, 14]. As an important category of plasmonic devices, plasmonic lenses based on thin metallic films were developed [15–21], being an alternative to the conventional dielectric-based refractive lenses. These nanostructured plasmonic lenses enable subwavelength focusing and allow all-optical or opto-electronic single-chip integration. They show great prospect for applications in many fields such as high-resolution imaging, single-molecular biosensing, optical data storage, and nanolithography. For example, plasmonics enables the totally new MLAs with the lens size of several micrometers to match the single pixel of the modern ultra-high-resolution CCD sensors. However, the nanostructured plasmonic lenses reported were found to reveal some shortcomings, e.g., the divergence of the transmitted optical field and the elaborate design of the nanostructures for focusing a specific wavelength.

Recently, the Odom research group reported a new type of metallic planar microlenses [22], the so-called patch structures consisting of a finite-sized nanohole array. These “patches” can focus the broadband white light with a little chromatic divergence. A batch fabrication method using the soft nanolithographic technique was also proposed to achieve a large number of such microlenses in parallel. However, the use of soft interference lithography (SIL) followed by a nanopatterning procedure including phase-shifting photolithography, etching, electron-beam deposition, and lift-off (PEEL), leads to the nanoholes on the periphery having a significantly smaller diameter than the central ones, and some are even blocked, causing a large deviation of the focal length from the design [23]. Meanwhile, the demonstrated MLA has a quite small fill factor (5 μm microlenses distributed in a 20-μm periodicity), which limits the optical throughput of the MLA. And no further discussions regarding the MLA are given.

In this paper, a kind of MLA with a relatively large fill factor is presented. The elemental microlens is similar to the Odom’s “patch” structure. Different from the other nanostructured plasmonic lenses as mentioned previously that require precisely designed nanostructures to operate for a specific wavelength, the metallic planar microlenses suggested here can focus single wavelengths of light across the entire visible spectrum as well as the broadband white light with little chromatic divergence. Because of the localized surface plasmon resonance in metallic nanostructures, the microlenses exhibit the enhanced transmittance at some specific wavelengths. Of particular interest here, the focusing coupling effect between two adjacent microlenses in an array is analyzed in detail. To the best of our knowledge, it has not been discussed in any other publications on the metallic planar microlenses, and we find that this effect plays a vital role when the diameter of microlenses and the periodicity are in the length scale of the wavelength, which is also rarely concerned for the conventional MLAs as their diameters are usually of hundreds of microns. How the focusing coupling effect influences the final focusing performance is shown by the numerical simulation method when the spacing between adjacent microlenses, the working wavelength, the diameter of microlenses, and the array size are changing. What is more, a low-cost, high-yield nanofabrication technique for achieving the microlens and its array is presented. This research provides an important technological reference for designing and fabricating the arrays of metallic planar microlenses to obtain a better control over their focusing performance.

## Methods

**Fig. 1:**
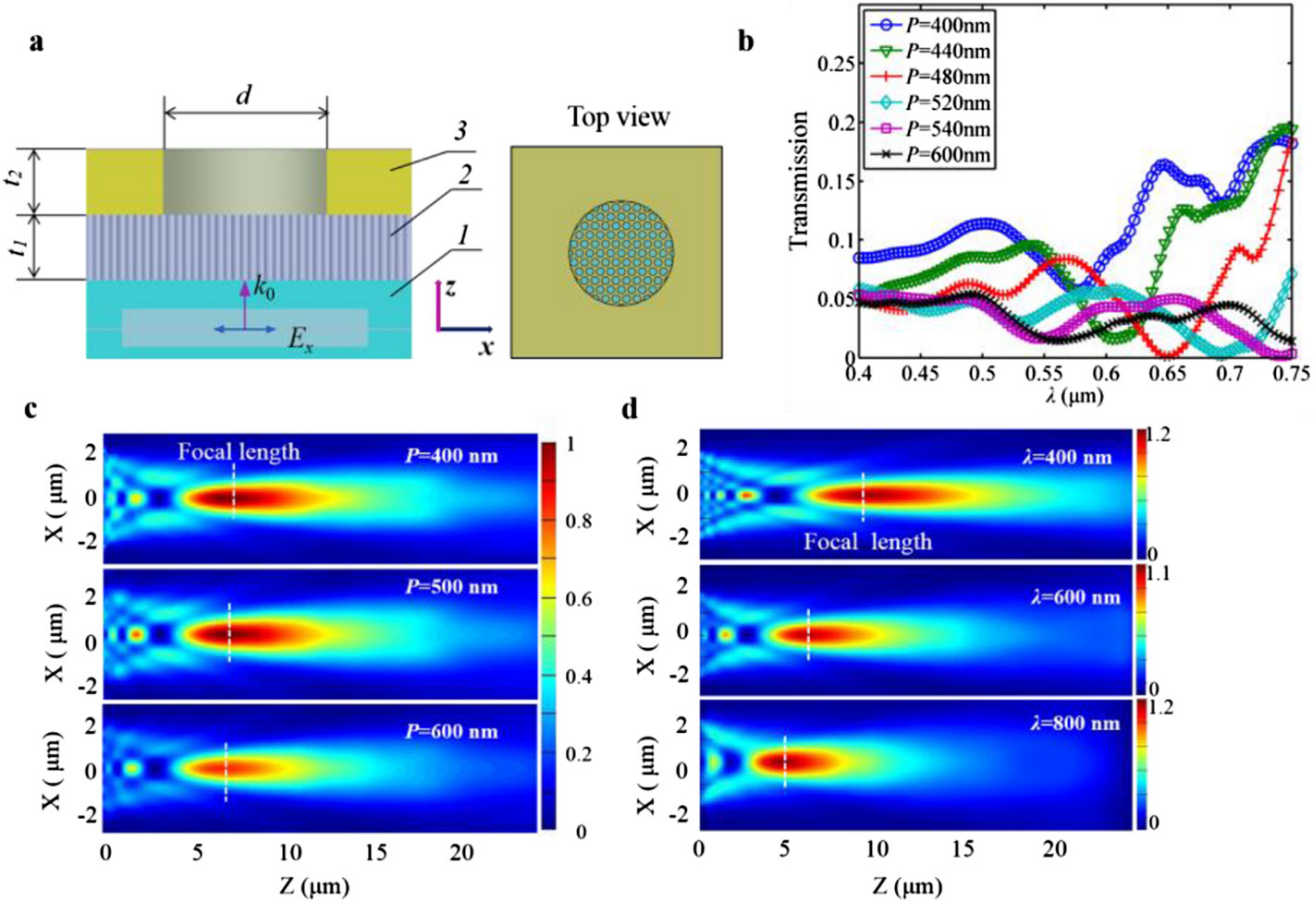
**a** Schematic of the designed broadband metallic planar microlens with *d* = 4 μm. *1* is the glass substrate, *2* is the first gold layer with a large-scale array of nanoholes, and *3* is the second gold layer defining the lens aperture. The *inset* gives the top view of the microlens. **b** The transmissive spectra of the lens when the periodicity *P* of nanoholes is varied from 400 to 600 nm. **c** 3D FDTD simulation results of the electric-field intensity for three cases of periodicity at the working wavelength of *λ* = 600 nm and the graphs are on the same color scale. **d** 3D FDTD simulation results of the electric-field intensity for three wavelengths when *P* = 400 nm

The designed metallic planar microlens is formed by an array of subwavelength nanoholes in the first gold film which is covered by a patterned micro-aperture film (the second gold film) to define the focusing features, as shown in Fig. 1a. The inset gives the top view of the microlens. Different from the square structural configuration as employed by the Odom group, our device has a triangular lattice. To this end, nanosphere lithography (NSL) technique can be purposely utilized to form a close-packed single-layer mask of nanospheres, for which the hexagonal structure is the most common circumstance [24, 25]. After modifying the diameter of nanospheres, the first gold layer is sputtered, and followed by the lift-off process to remove the nanospheres, and a large-scale nanohole array is thus achieved. Then, the second gold film is patterned on the first holey gold film with the desired lens size, which dominates the focusing performance of the ultimately fabricated microlenses. In addition to the gold, other metallic materials can also be adopted, and different types of metals for the two layers are also possible, all depending on the specific applications.

Figure 1a gives the analytical model for the designed metallic planar microlenses, and a full-wave three-dimensional (3D) electromagnetic simulation was performed by using the finite-difference time-domain (FDTD) method. Perfectly matched layer was applied to all the outermost borders and the mesh size was set to 15 × 15 × 15 nm^3^. The value of permittivity and permeability for the dispersive gold material is taken from the Chemical Rubber Company (CRC) data sheet [26]. The whole structure was normally illuminated by a linearly polarized plane wave with the wave vector *k*_0_ in the *z* direction, and the electric field is polarized along the *x* direction.

## Results and Discussion

### Focusing Performance of an Isolated Microlens

The simulated microlens had an aperture size of 4 μm, and the diameter of nanoholes was 300 nm. The two gold layers both had a thickness of 100 nm. Figure 1b gives the simulation results of the transmissive spectra of the lens when the periodicity *P* of nanoholes is varied from 400 to 600 nm. The derived maximum light transmission is about 20 % for the periodicity less than 500 nm, all appearing near the working wavelength of *λ* = 0.75 μm. According to the classical Bethe theory, the transmission efficiency *η*_*B*_ (normalized to the aperture area) can be derived by *ηB* = 64(*kr*)^4^/27*π*^2^ [27]. As a result, the calculated transmission is only 1.89 %, and an extraordinary optical transmission (EOT) phenomenon is evident, contributing significantly to a high-efficiency focusing. Figure 1c presents the simulation results of the electric-field intensity cutting through the focal plane for the typical three periodicities. The focal spot is formed by the interference of in-phase waves which diffract from the nanoholes. Since the EOT resonance can be tuned by changing the periodicity of nanoholes, the resonances present at different wavelengths, as seen in Fig. 1b. This not only provides a means to control the optical throughput of the microlenses at specific wavelengths, but also ensures that microlenses can be easily designed with a high-efficiency focusing. However, the periodicity of nanoholes reveals an insignificant influence on the focal length, depth of focus (DOF), and full-width at half-maximum (FWHM) of the focal spot. The focal length depends mainly on the lens size and the working wavelength. The wavelength has a large impact on the focusing performance, which can be clearly seen in Fig. 1d. Normally, the smaller the working wavelength is, the larger the focal length is.

**Fig. 2:**
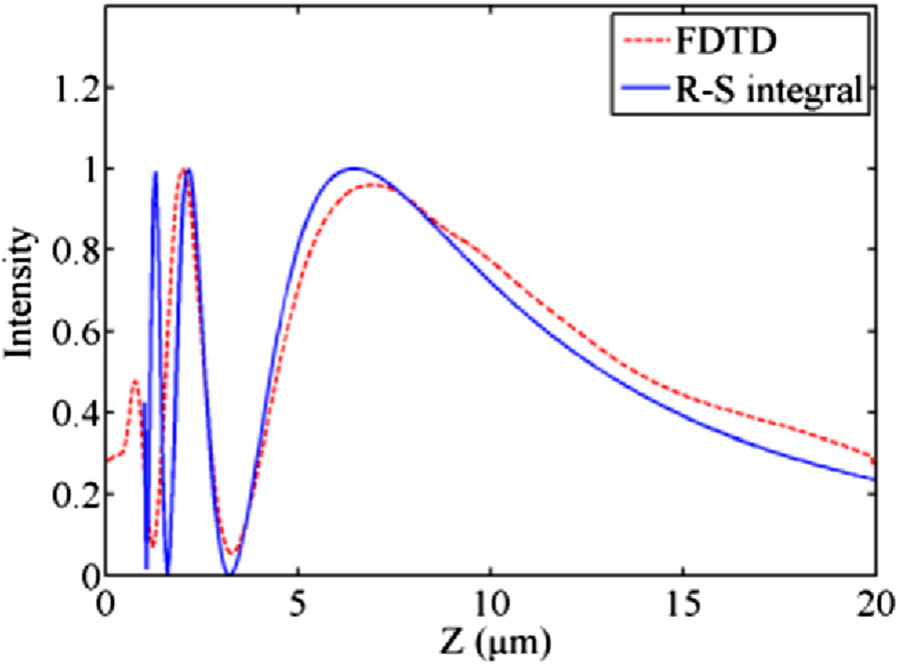
The axial light intensity of the calculated R-S integral and the FDTD simulation for the designed metallic planar microlens. The results show a good agreement on the focusing performance (*ρ* = 2 μm, *λ* = 600 nm)

The axial light intensity of the lens (along the *z* direction) can be predicted by the Rayleigh-Sommerfeld (R-S) integral under the paraxial approximation [28, 29], expressed as

1 $$ I\left(0,Z\right)=4A{\left[ \sin \left(\frac{\pi {\rho}^2}{2\lambda Z}\right)\right]}^2 $$

where *I* is the light intensity, *A* is the maximum intensity, *ρ* is the radius of the lens, and *Z* is the distance away from the lens. Accordingly, the maximum light intensity of *I*, indicating the location of the focal spot, can be achieved at a position of *Z*_*m*_ = *ρ*^2^/*λ*, well verifying the previous analysis. Figure 2 compares the simulation and calculation results for the light intensity along the *z* direction, from which we can see that both results show a good agreement.

### Focusing Coupling Effect for the Arrayed Microlenses

Generally, as for the traditional microlens arrays, there is no obvious coupling between two adjacent microlenses as the spacing and the lens size are much larger than the working wavelength, in which case the diffraction effect is ignorable. However, when the spacing and the lens size approach to the length scale of the wavelength, the diffraction of light does exist and produces a significant impact on the final focusing optical field. Therefore, in order to have a good control over the focusing performance of the arrays consisting of micro-sized metallic planar microlenses, the coupling effect between two adjacent microlenses needs to be considered during the design of such microlens arrays.

#### The Spacing between Two Adjacent Microlenses

**Fig. 3:**
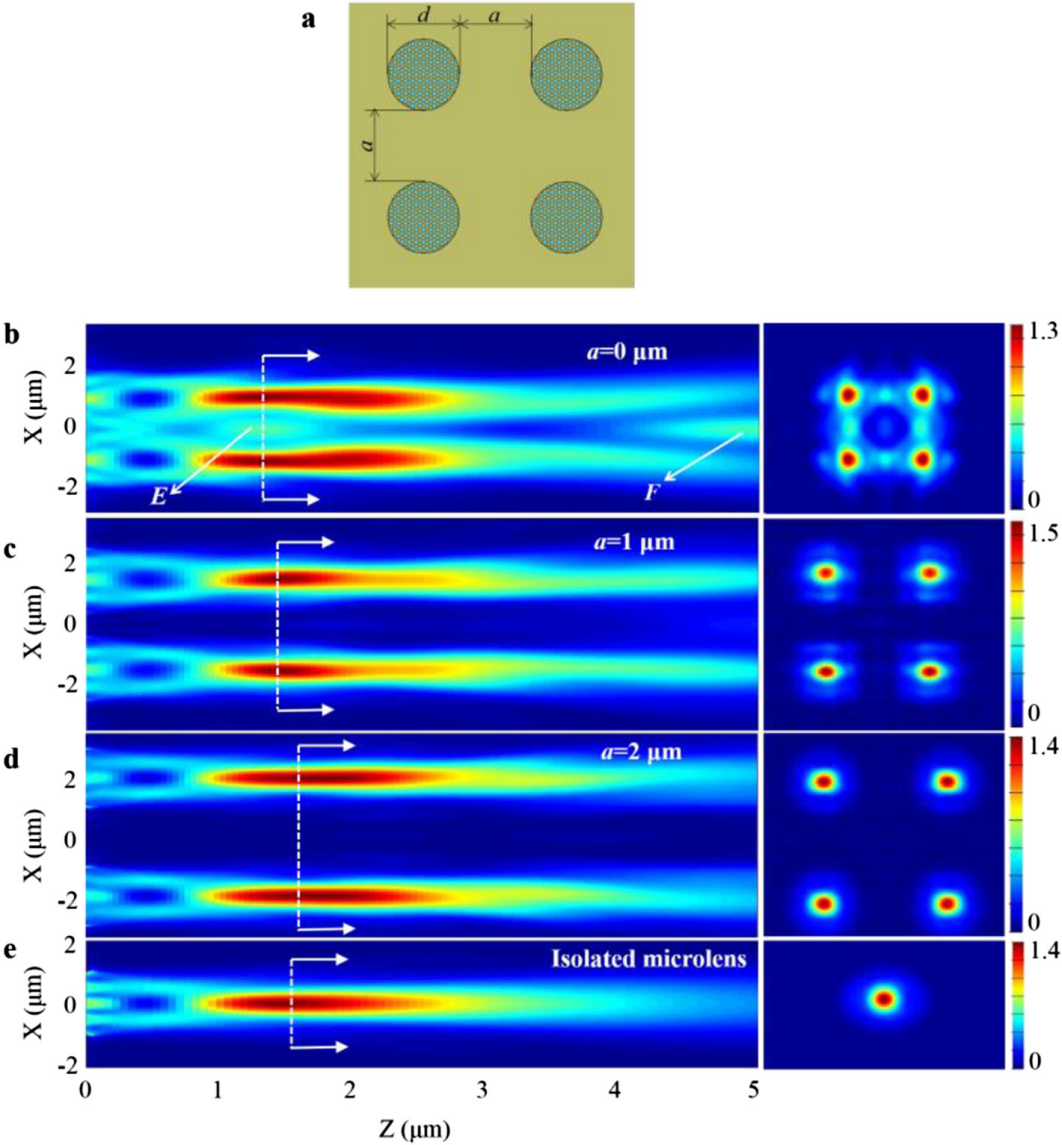
**a** The top view of the 2 × 2 microlens array. Simulation results of the electric-field patterns of 2 × 2 microlens array (*d* = 2 μm) parallel to the *xz* plane and the focal planes for the 600 nm working wavelength, corresponding to a different spacing of **b** 0 μm, **c** 1 μm, and **d** 2 μm. The coupling effect between two adjacent microlenses put side by side (*a* = 0 μm), as denoted by the coupling points *E* and *F*, can be clearly observed. **e** Electric-field pattern of an isolated microlens

First, as shown in Fig. 3a, we construct a typical 2 × 2 microlens array with the spacing *a* varying from 0 to 4 μm to explore its focusing properties (*d* = 2 μm, *λ* = 600 nm). The simulation results of the electric-field patterns parallel to the *xz* plane through the focal spots for three typical cases of *a* = 0, 1, 2 μm are shown in Fig. 3b–d, respectively, together with the electric-field patterns along the focal planes. For comparison, the focusing electric-field pattern of an isolated microlens is also given in Fig. 3e. As we can see, the coupling effect seems obvious for the spacing of 0 and 1 μm, especially for the former case, in which two coupling points *E* and *F* appear, *E* close to the focal spots and *F* far away from the focal plane. The focal points are modulated and become asymmetric in the *x* and *y* directions. We think the electric-field component of the incident plane wave initially defined leads to it. As the spacing increases, the focusing optical field for a single microlens in an array gradually accords with the isolated lens, meaning that the coupling effect is disappearing.

**Fig. 4:**
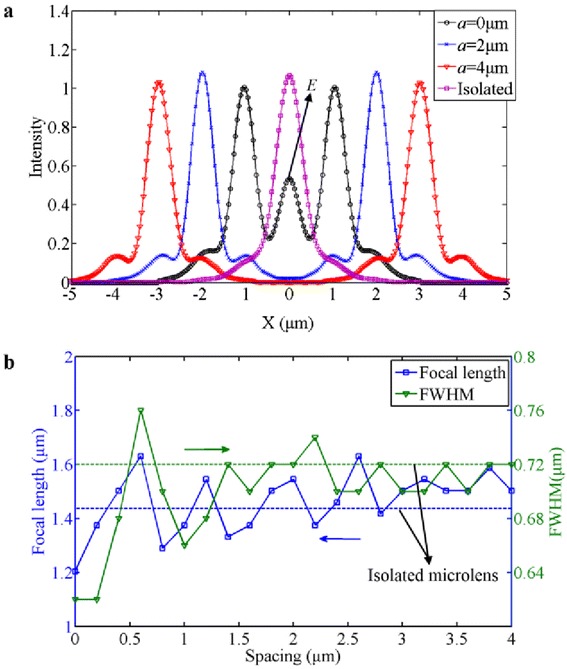
**a** The line-scanning profiles of light intensity across the focal spots of the 2 × 2 microlens array with the spacing of *a* = 0, 2, 4 μm, accompanied by the result of the isolated microlens. The coupling point *E* for the case of *a* = 0 μm can be clearly observed. **b** The derived focal length and FWHM of the focal spots as the spacing changes. The *blue* and *green dashed lines* represent the focal length and FWHM of the isolated microlens

Figure 4 gives the achieved line-scanning profiles of light intensity across the focal spots of the 2 × 2 microlens array, and also the extracted focal length and FWHM of the formed focal spots. In Fig. 4a, the coupling point *E* for the case of *a* = 0 μm is obvious. However, for the cases of *a* ≥ 2 μm, the coupling effect disappears to a negligible level. In Fig. 4b, when the spacing *a* < 1 μm, the fluctuation of the focusing properties is of tremendous magnitude. However, as the spacing gradually increases, it approaches to the focusing properties of the isolated lens, as illustrated by the blue and green dashed lines, which gives a good demonstration to show the influence of the lens spacing on the focusing performance.

The derived results for all the cases of different spacings are listed and compared in Table 1, including the focal length, FWHM, DOF, and the maximum light intensity *I*_max_. The incident light intensity is set to 1 here, and the *I*_max_ in Table 1 is scaled to the incident intensity. Although the realized focal length of the isolated microlens is 1.51 μm, agreeing with the theoretical value of 1.60 μm, the actual focal length of the microlenses in a 2 × 2 array with the spacing of 0 μm is changed to 1.21 μm, resulting in a prominently large deviation from the original design.

**Table 1:** Derived focusing performance for different cases of the lens spacing

Spacing *a* (μm)	Focal length (μm)	FWHM (μm)	DOF (μm)	*I*_max_ (a.u.)
0	1.204	0.62	1.919	1.226
1	1.375	0.66	2.090	1.544
2	1.545	0.72	2.559	1.395
3	1.545	0.70	2.132	1.435
4	1.503	0.72	2.474	1.349
Isolated lens	1.509	0.72	2.317	1.374

According to the above analysis, a large spacing is beneficial for realizing the highly directional beaming with the independent foci of microlenses in an array. However, the large spacing results in a relatively low fill factor, which deteriorates the optical efficiency. For practical applications, a trade-off should be taken.

#### The Working Wavelength

**Fig. 5:**
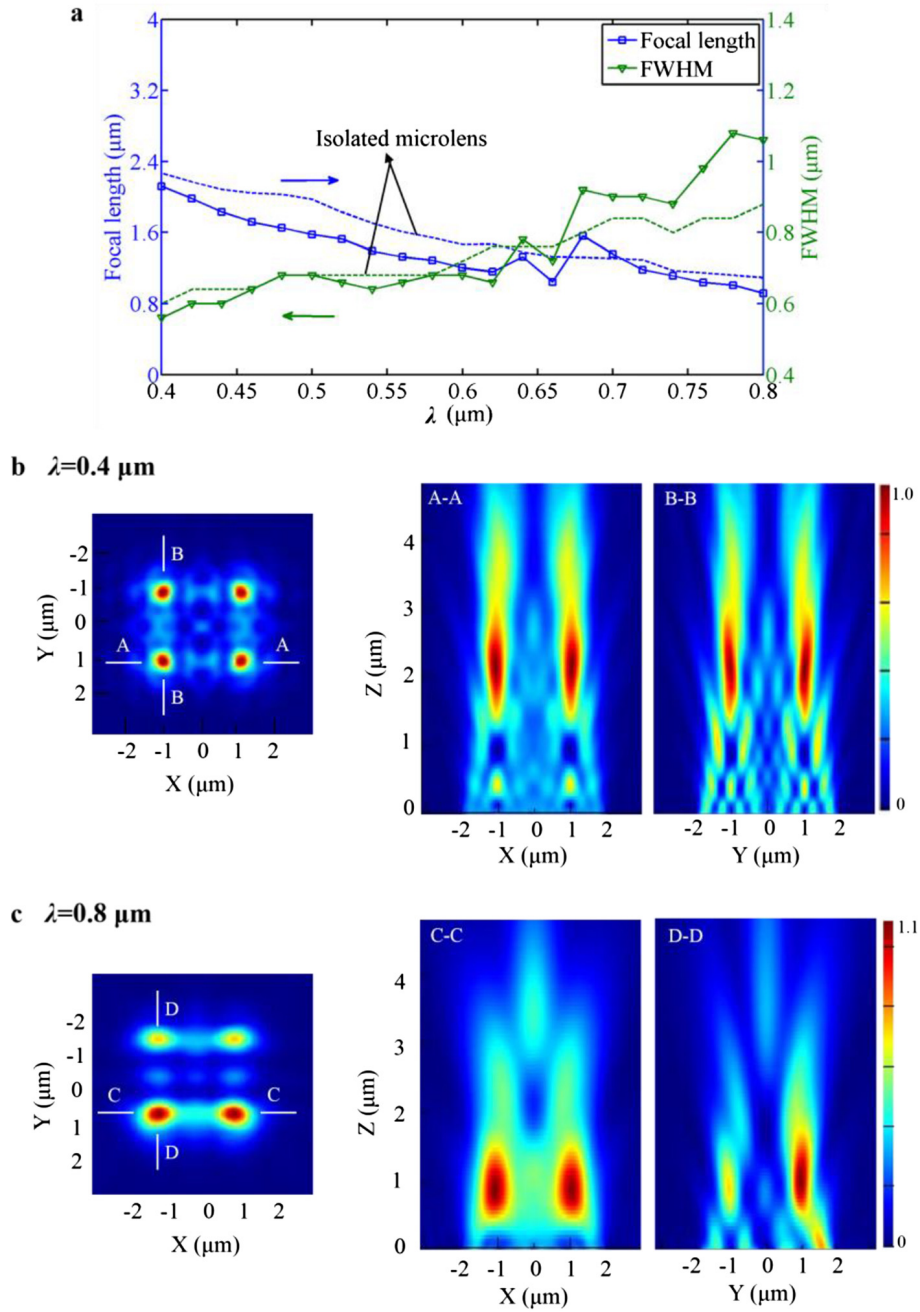
**a** Simulation results of the focal length and FWHM of the microlenses in a 2 × 2 array with the working wavelength *λ* changing over the whole visible spectrum (*d* = 2 μm, *a* = 0 μm). **b** 3D FDTD simulated electric-field intensity of the focal plane of the microlens array for *λ =* 0.4 μm and the focusing patterns of *A*–*A* and *B*–*B* cross sections. **c** The simulated electric-field intensity of the focal plane for *λ =* 0.8 μm and the focusing patterns of *C*–*C* and *D*–*D* cross sections

Second, the influence of the working wavelength on the focusing performance of the microlens array is also investigated for the same array size as mentioned above. Because of the strong coupling at a small spacing, the spacing is set to zero in order to facilitate the analysis of the focusing coupling. Figure 5a presents the simulation results of the focal length and FWHM of the microlenses with the working wavelength *λ* changing over the whole visible spectrum. As the working wavelength increases, the focal length has a general trend of decreasing, and the FWHM reveals an increasing trend. What puzzles us is that for the working wavelengths between 640 and 680 nm, there exist singular points to complicate the analysis for both the focal length and FWHM. The underlying physical explanation needs a further survey.

From Fig. 5a, we can deduce a general rule that as the working wavelength increases, the coupling effect become more apparent, e.g., for the case of *λ* = 0.4 μm, the deviation of the focal length in contrast to the isolated lens is 6.7 %, compared to the 17.3 % for the case of *λ* = 0.8 μm. Furthermore, the polarization of the incident light also seems to affect the coupling effect. Figure 5b, c presents the focal planes and the electric-field patterns along the polarization direction (*xz* plane) and the direction perpendicular to it (*yz* plane) for *λ* = 0.4 and *λ* = 0.8 μm, respectively. The results indicate that a stronger coupling appears at *λ* = 0.8 μm where the polarization impacts the focusing evidently.

#### The Diameter of Microlenses

**Fig. 6:**
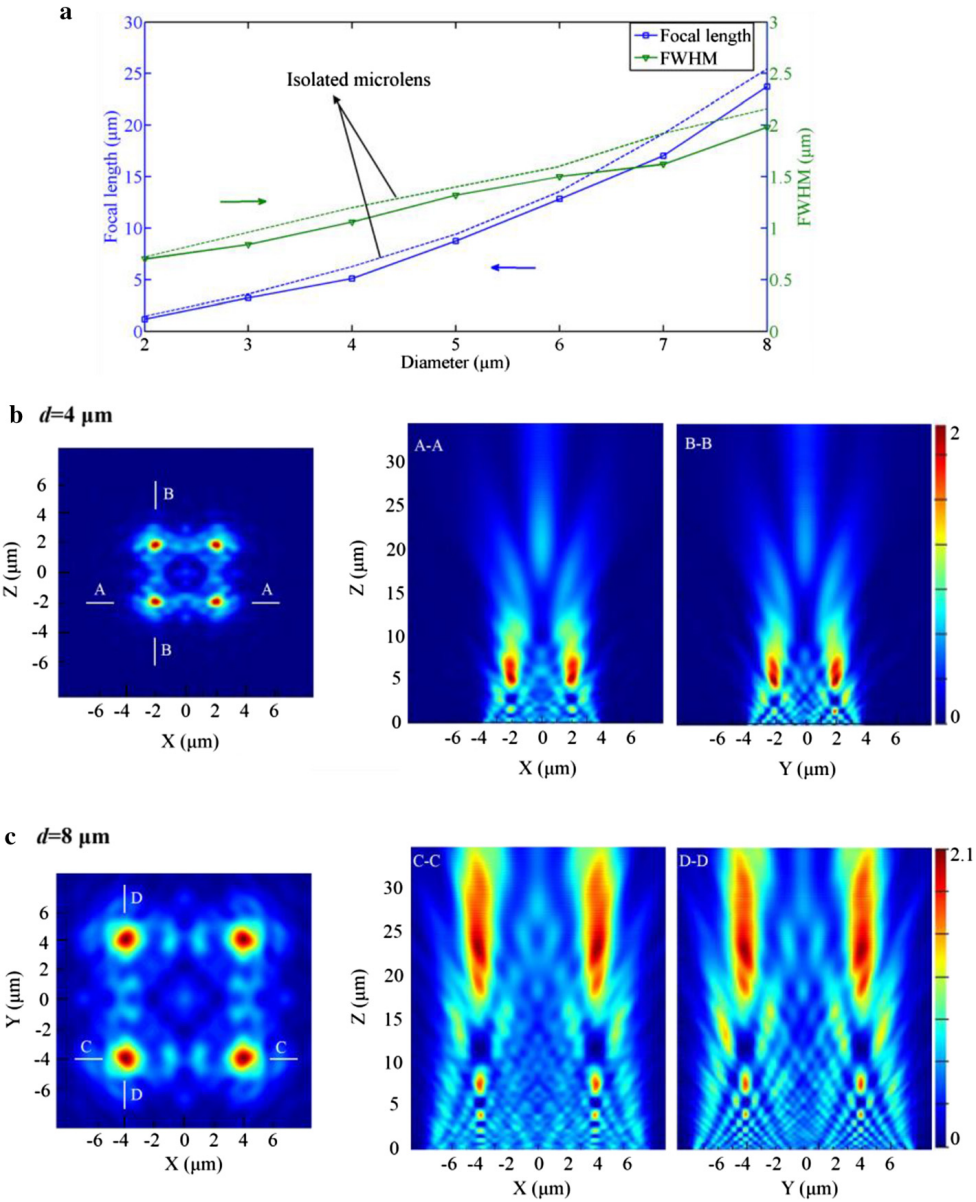
**a** Simulation results of the focal length and FWHM of the microlenses in a 2 × 2 array as the diameter of lenses varying from 2 to 8 μm (*a* = 0 μm, *λ* = 0.6 μm). **b** The simulated electric-field intensity of the focal plane of the microlens array for *d =* 4 μm and the focusing patterns of *A*–*A* and *B*–*B* cross sections. **c** The simulated results of the focal plane for *d =* 8 μm and the focusing patterns of *C*–*C* and *D*–*D* cross sections

Figure 6 shows how the diameter of microlenses affects the focusing performance when they are configured in an array. Similar to the isolated microlens, both the focal length and FWHM of the microlenses in the array increase as the diameter gets larger. Due to the coupling effect, the focal length and FWHM of microlenses in the array are both slightly less than those of the isolated lens. The general rule derived is that the coupling effect becomes weaker when the diameter of microlenses is enlarged, e.g., the deviation of focal length of 20.4 and 6.7 % corresponding to the diameter of 2 and 8 μm, respectively.

The focal planes and the electric-field patterns along the polarization direction (*xz* plane) and the direction perpendicular to it (*yz* plane) for *d* = 4 and 8 μm are shown in Fig. 6b, c, respectively, from which we can see that the polarization plays an insignificant role for the final focusing performance in this situation. As a result, for a good prediction over the focusing performance, a larger diameter of microlenses is preferred, explaining well the reason that there is no coupling appearing for the conventional MLAs as reported.

#### The Array Size

**Fig. 7:**
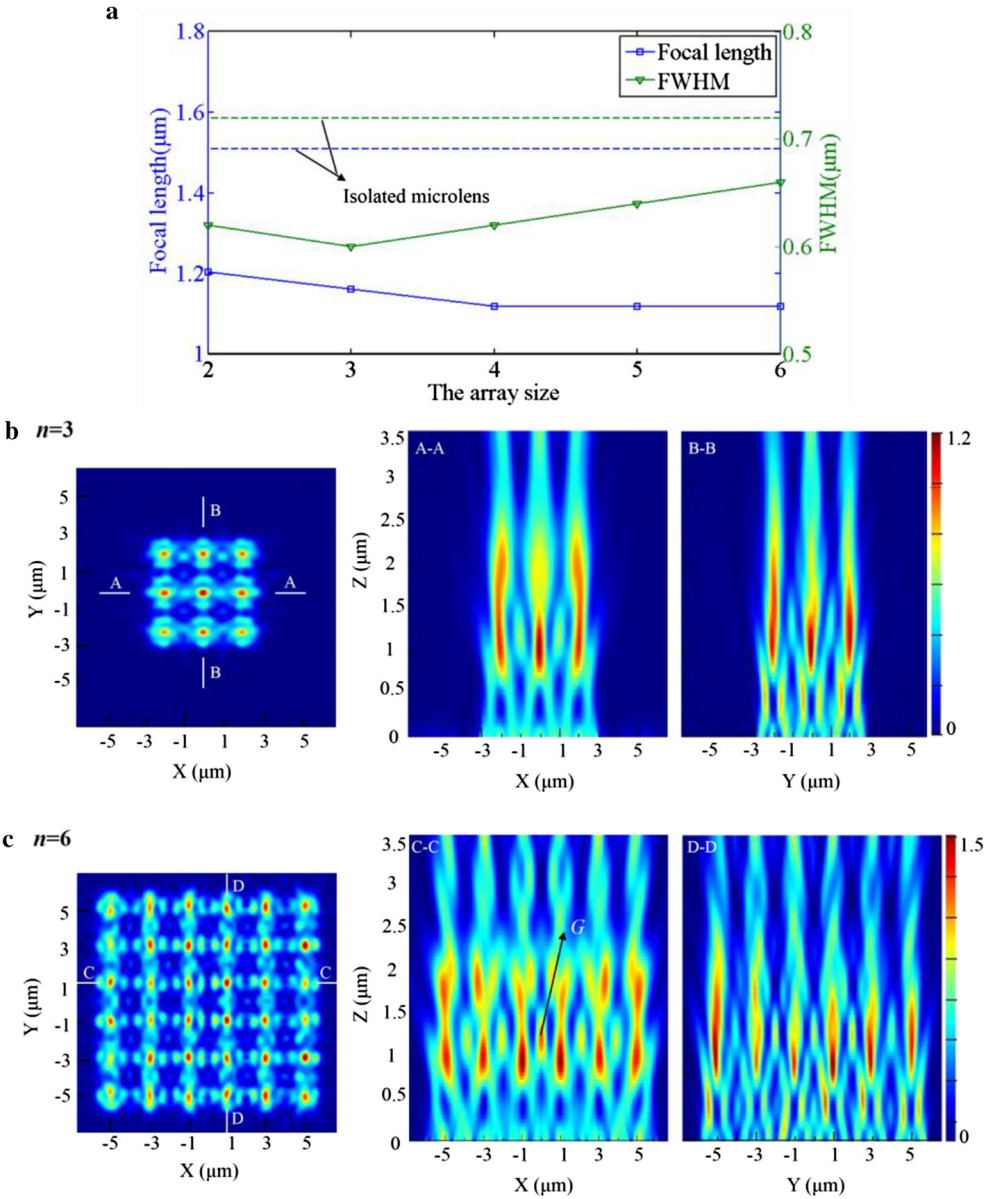
**a** Simulation results of the focal length and FWHM of the microlenses located in the geometrical center of the array with the scale varying from 2 × 2 to 6 × 6 (*a* = 0 μm, *λ* = 0.6 μm, *d* = 2 μm). **b** The simulated electric-field intensity of the focal plane of the microlens array for *n =* 3 and the focusing patterns of *A*–*A* and *B*–*B* cross sections. **c** The simulated results for *n =* 6 and the focusing patterns of *C*–*C* and *D*–*D* cross sections

Finally, a larger array size is investigated, meaning from the 2 × 2 array changed to 6 × 6 array, which may be of more importance for the practical applications. Figure 7a gives the simulation results of the focal length and FWHM of the microlenses located in the geometrical center of the array when the scale varies, while keeping the diameter of microlenses of 2 μm. The focal length of the arrayed microlenses at *λ* = 600 nm decreases slightly from 1.21 to 1.12 μm, while that of the isolated lens is 1.51 μm (Fig. 7a). This deviation is induced by the stronger coupling as the array size increases. The added microlenses make the coupling occur not only between two adjacent lenses but also between the interval lenses. Figure 7b, c presents the simulation results of the focal planes and the electric-field patterns for the cases of 3 × 3 and 6 × 6 microlens array in the two perpendicular planes. It is evident that a clear difference exists due to the strong polarization-related coupling.

Another point worthy to note is that the intensity of the central coupling point *G*, as indicated in Fig. 7c, is higher than the other coupling points, which is caused by the superimposed coupling of microlenses in the array. Furthermore, the focal spots created by the peripheral microlenses are distinctly asymmetric with a weak light intensity, while the inner ones surrounded by other microlenses are almost symmetric, as well as having a stronger light intensity. This fringing effect makes the accurate control over the focusing performance of microlenses in an array even more complicated.

## Conclusions

In summary, we suggest a kind of metallic planar microlenses, consisting of finite-sized two-dimensional nanohole arrays with a triangular lattice distribution. When the lens size and the spacing between two adjacent microlenses become comparable to the working wavelength, the diffraction effect cannot be ignored anymore, which is normally out of consideration for the conventional microlens arrays. Based on the FDTD numerical simulation method, the focusing performance of the microlenses in an array is investigated in detail as the spacing between adjacent microlenses, the working wavelength, the diameter of microlenses, and the array size are changing. The results show that a larger spacing, a larger lens size, a shorter wavelength, or a smaller array scale can lead to a weaker focusing coupling effect. This research provides a valuable reference to design an array of metallic planar microlenses and to have a good control over its focusing performance. Nevertheless, a further study on this topic is needed to verify the simulation results by the experiments, and also some other problems like the polarization-related coupling and the fringing effect of the focusing remain to be investigated further. The broadband focusing capabilities and the miniaturization of microlenses and their arrays proposed opens a great potential for such applications as high-resolution imaging, flat panel displays, beam collimating for optical fibers, and high-efficiency solar cells.

## Abbreviations

CCDs: charge-coupled devices
DOF: depth of focus
EOT: extraordinary optical transmission
FDTD: finite-difference time-domain
FWHM: full-width at half-maximum
MLAs: microlens arrays
NSL: nanosphere lithography
SIL: soft interference lithography
